# C-reactive Protein as a Negative Predictor of Anastomotic Leak Following Elective Colorectal Resection: A Beacon of Assurance?

**DOI:** 10.7759/cureus.74156

**Published:** 2024-11-21

**Authors:** Ali Yasen Mohamedahmed, Chinedu Ndegbu, Kapil Agrawal, Sreedutt Murali, Shumaila Tanveer, Sanaa Elgaddal

**Affiliations:** 1 Department of Colorectal Surgery, The Royal Wolverhampton NHS Trust, Wolverhampton, GBR

**Keywords:** anastomosis leak, colorectal anastomosis, colorectal surgery, c-reactive protein, postoperative outcomes

## Abstract

Background

To evaluate the accuracy and optimal C-reactive protein (CRP) level for detecting anastomotic leak (AL) in patients following elective colorectal resection.

Methods

A retrospective data collection of patients undergoing elective colorectal resection with primary anastomosis at a single institution was performed. Data were collected between June 2021 and November 2022. All colorectal resections and any anastomosis type were included. The following information was collected: basic patient demographics, operative and postoperative complications, and daily CRP results for the first seven postoperative days (POD). SPSS version 27 (IBM Corp., Armonk, NY) was used for all data analysis.

Results

A total of 231 patients with a mean age of 68.8 ± 14.4 years were included. The most common surgical procedure was a right hemicolectomy (46.3%), followed by anterior resection (42.8%), segmental colectomy (7.4%), and subtotal colectomy (3.4%). The overall AL rate for this cohort was 3%, and the median length of hospital stay was six days (mean: 7.6 ± 5.1 days). POD3, POD4, and POD5 showed an area under the curve of 0.73 (P = 0.07), 0.90 (P = 0.001), and 0.95 (P = 0.002), respectively. An optimal CRP cut-off value of 160 mg/L on POD4 resulted in a sensitivity of 85%, specificity of 83%, negative predictive value of 98%, and positive predictive value of 24%.

Conclusion

CRP is an excellent negative predictor of AL following colorectal resection and primary anastomosis. Patients with a POD4 CRP of <160 mg/L may be earmarked for hospital discharge if clinically appropriate.

## Introduction

Major colorectal resections can be challenging and are associated with peri-operative complications [1]. Anastomotic leak (AL), with an estimated incidence of up to 30%, is the most dreaded and frequently cited complication following colorectal surgery [1-4]. Moreover, morbidity rates of 35% [5] and mortality rates ranging from 1% to 16% make colorectal resections with primary anastomosis high-risk procedures [5,6]. Consequently, these complications translate into a greater utilisation of resources, increase the length of hospital stay, and have significant cost implications [3].

The conventional remedial treatment of AL relies on the early detection of clinical signs, including fever, abdominal pain, peritoneal or faecal drain output, and prolonged ileus [1-3,6]. Enhanced Recovery After Surgery (ERAS) pathways and guidelines have helped standardise the peri-operative care of such patients. The development and use of these protocols have been credited with reducing the significant morbidity associated with major colorectal resections [3].

Studies have attempted to establish early detection pathways for recognising AL. Various biomarkers such as C-reactive protein (CRP), procalcitonin (PCT) levels, interleukins, white blood cells, and their association with AL have been investigated [1-4,7]. The most commonly used clinically is CRP (acute phase protein), which is often closely correlated with the development of an AL [2,6,8].

Moreover, CRP level was considered an accurate negative predictor for detecting AL following oesophagogastric surgery [9]. The Italian Colorectal Anastomotic Leakage (iCral) study group reported CRP to be an effective negative predictor of AL, and the addition of PCT levels further reinforced the diagnosis and the negative predictive value [1]. However, considering the cost factor in performing regular PCT levels for this cohort of patients, a recent meta-analysis by Cousin et al. found no additional benefit in adding PCT levels [10].

We evaluated the accuracy and optimal CRP cut-off value for AL in patients undergoing major elective colorectal resection at our institution.

## Materials and methods

This is a retrospective cohort study and was approved by the Audit Department at the Royal Wolverhampton NHS Trust (Approval Number: 16294). All patients who underwent elective colorectal resections with primary anastomosis during June 2021 and November 2022 were included. All colorectal resections (right hemicolectomy, segmental colectomy, subtotal colectomy, anterior resection) and anastomosis types (ileocolic, colocolic, and colorectal) were included. Our exclusion criteria were patients under 18 years of age, patients who received an end stoma (no anastomosis), patients who had emergency procedures, and patients whose CRP results were unavailable for ≥ three days out of the first seven postoperative days (PODs).

Prophylactic antibiotics, bowel preparations, diverting stomas, and drain placement were decided according to the operating surgeon’s preference. AL was suspected clinically if the patient developed peritonitis, sepsis, and high inflammatory markers, and further confirmed with a computed tomography (CT) scan in all patients.

The following information was collected and recorded in an Excel spreadsheet (Microsoft Corporation, Redmond, WA) after being retrieved from the hospital's electronic system: patient demographics, indication for surgery, intra-operative details, postoperative complications, and daily CRP results for the first seven PODs. SPSS version 27 (IBM Corp., Armonk, NY) was used for all data analysis. Categorical demographic and clinical characteristics were described as frequency (percentage). In addition, continuous variables were shown as mean with standard deviation or median and range.

The mean CRP of POD1 through to POD7 was calculated and constructed in a graph according to the development of an AL and surgical approach (open surgery, laparoscopy, and robotic platform). Moreover, receiver operating characteristic (ROC) curves were plotted for AL's mean CRP of POD 1-5. Sensitivity, specificity, negative predictive value (NPV), positive predictive value (PPV), accuracy, and area under the curve (AUC) were calculated.

## Results

A total of 231 patients who underwent elective colorectal surgery and primary anastomosis between June 2021 and November 2022 were included. The mean age of the cohort was 68.8 ± 14.4 years, with a male-to-female proportion of 57.6% and 42.4%, respectively. The primary indication for surgery was colorectal cancer in the majority of cases (86.5%), followed by inflammatory bowel disease (5.6%), diverticular disease (3.9%), polyps (3.5%), and sigmoid volvulus (0.4%).

Right hemicolectomy was performed in 46.3% of patients, anterior resection in 42.8%, segmental colectomy in 7.4%, and other types of colectomies in 3.4%. The surgical approach was laparoscopic in 39.8%, open surgery in 30.8%, robotic in 28.6%, and laparoscopic converted to open in 0.8% of cases.

A de-functioning stoma was created in 24.7% of patients, and a stapling device was used for most primary anastomosis (84.8%). The median length of hospital stay was six days, with a mean of 7.6 ± 5.1 days. Characteristics of the included patients are outlined in Table 1.

**Table 1:** Baseline characteristics and outcomes of the included patients. n: total number of patients; SD: standard deviation; ASA: American Society of Anesthesiologists.

Variables		Results, n (%) (Total n = 231)
Age (mean ± SD)		68.8 ± 14.4
Gender	Female	98 (42.4)
	Male	133 (57.6)
ASA grade	I	7 (3)
	II	146 (63.2)
	III	78 (33.8)
Indication for surgery	Colorectal cancer	200 (86.5)
	Inflammatory bowel disease	13 (5.6)
	Diverticular disease	9 (3.9)
	Polyps	8 (3.5)
	Sigmoid volvulus	1 (0.4)
Neo-adjuvant therapy	chemotherapy	40 (17.3)
	Chemo-radiotherapy	12 (5.2)
Procedure	Right hemicolectomy	107 (46.3)
	Segmental colectomy	17 (7.4)
	Anterior resection	59 (42.8)
	Other types of colectomies	8 (3.4)
Surgical access	Laparoscopic/laparoscopic assisted	92 (39.8)
	Laparoscopic converted to open	2 (0.8)
	Open	71 (30.8)
	Robotic	66 (28.6)
De-functioning stoma		57 (24.7)
Anastomosis technique	Stapler	196 (84.8)
	Hand-sewn	35 (15.2)
Anastomosis leak		7 (3)
Surgical site infection		9 (3.8)
Respiratory complications		8 (3.4)
Abdominal collection		4 (1.7)
30-day mortality		1 (0.4)
Length of hospital stay (mean ± SD)		7.57 ± 5.1

**Figure 1:**
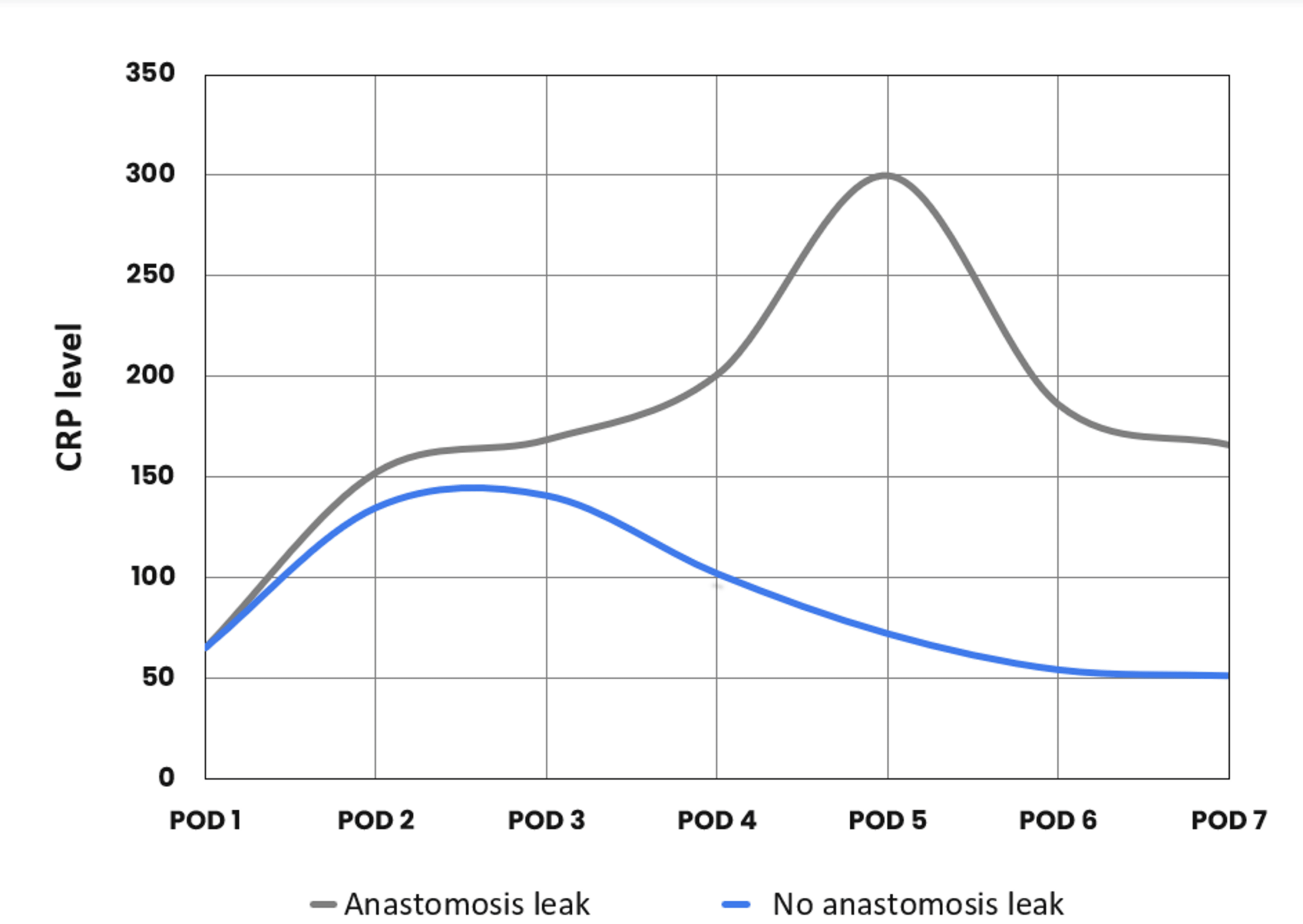
Relation between C-reactive protein (CRP) and anastomosis leak and no anastomosis leak. POD: postoperative day.

Our cohort had an AL rate of 3% (n = 7). Of these, four patients were treated surgically, and three with interventional radiology procedures and conservative management. A comparison of mean CRP levels for POD1 to POD7 between the AL group and no AL group showed comparable results in the first three PODs. However, the AL group's mean CRP level continued to climb from POD4 whilst steadily reducing in the no AL group (Figure 1).

**Figure 2:**
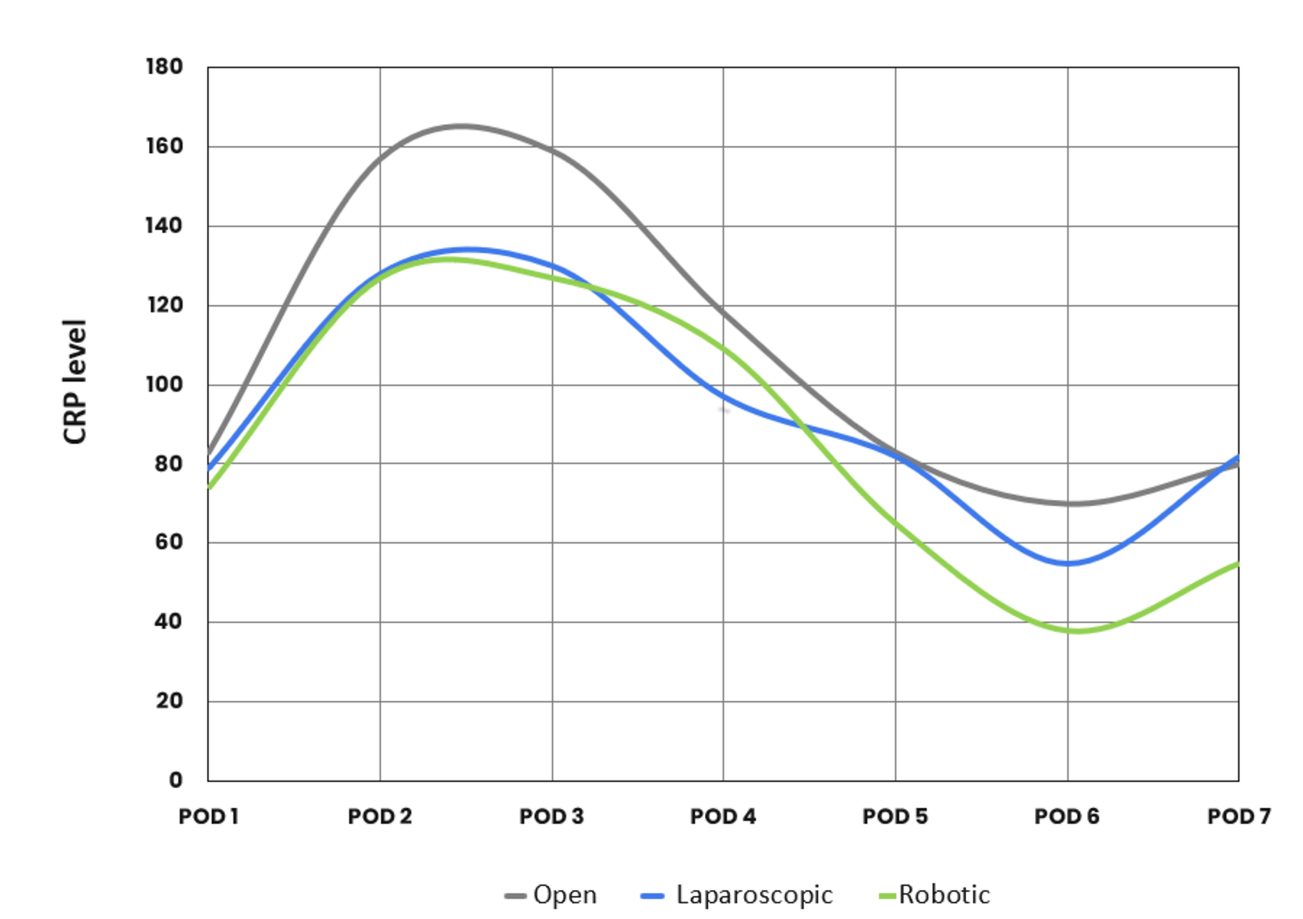
Relation between C-reactive protein (CRP) and surgical approach (open, laparoscopic, and robotic). POD: postoperative day.

Additionally, in the first three PODs, the mean CRP levels of patients undergoing open surgery were higher than those undergoing minimally invasive surgery (Figure 2).

**Figure 3:**
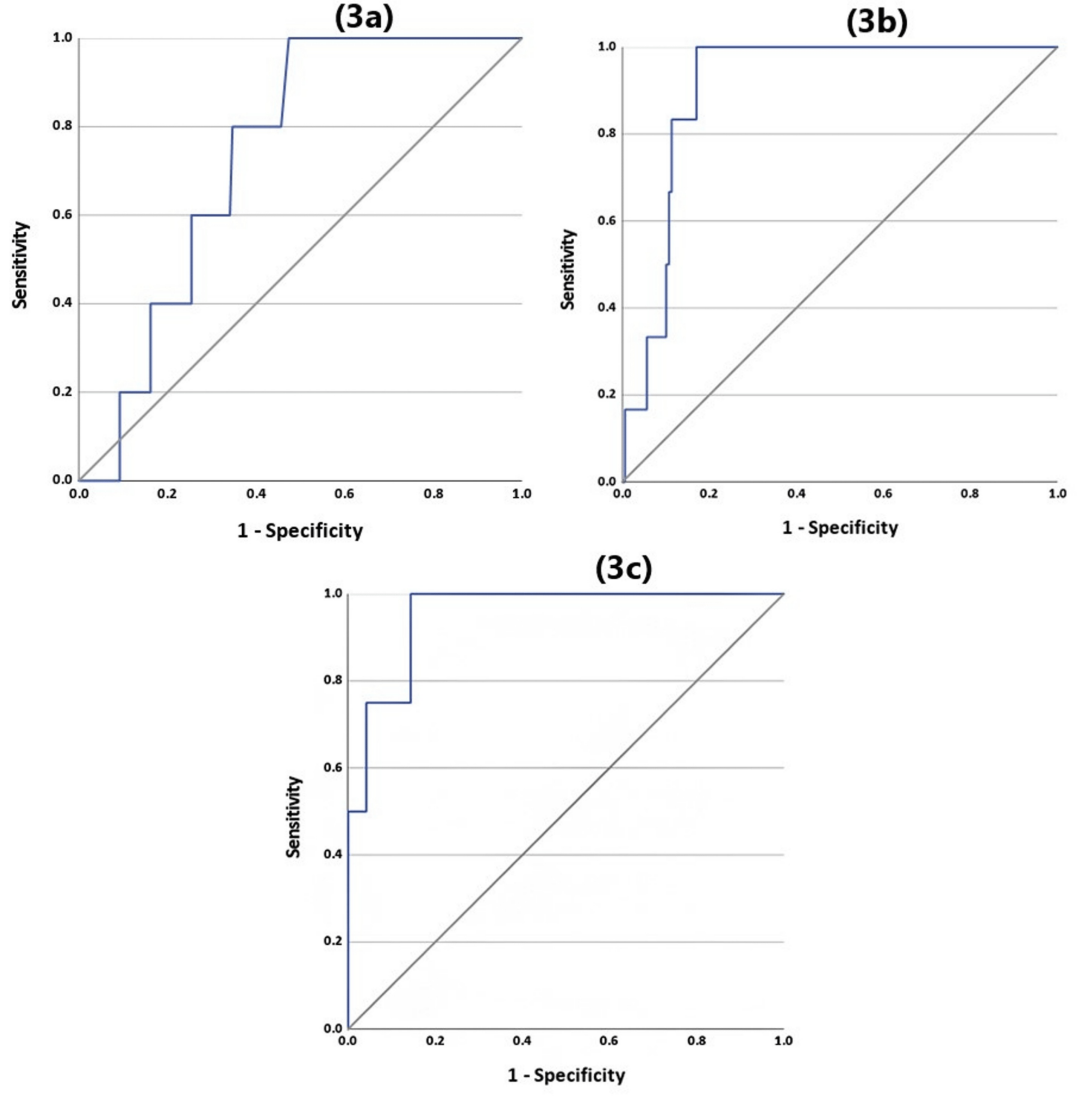
Receiver operator characteristics (ROC) curve for C-reactive protein's relation to anastomosis leak. Blue line indicates the test. 3a, 3b, and 3c represent POD3, POD4, and POD5, respectively. POD: postoperative day.

The ROC analysis was conducted to evaluate the association between CRP levels and AL. CRP levels on POD3, POD4, and POD5 showed an AUC of 0.73 (P = 0.07), 0.90 (P = 0.001), and 0.95 (P = 0.002), respectively (Figure 3 and Table 2).

**Table 2:** Receiver operator characteristics (ROC) curve analysis results for the association between anastomosis leak and C-reactive protein. POD: postoperative day; AUC: area under the curve.

Parameter	POD 1	POD 2	POD 3	POD 4	POD 5
AUC	0.53	0.59	0.73	0.90	0.94
P-value	0.81	0.41	0.07	0.001	0.002

Notably, a CRP cut-off value of 160 mg/L on POD4 yielded an 85% sensitivity and an 83% specificity for AL. The NPV at this threshold was 98%, and the PPV was 24% (Table 3).

**Table 3:** Specificity, sensitivity, accuracy, positive predictive value (PPV), and negative predictive value of C-reactive protein (CRP) of 160 mg/l for anastomosis leak. POD: postoperative day.

POD	Specificity	sensitivity	Accuracy	PPV	NPV
POD 3	0.80	0.65	79.6%	17.5%	97.4%
POD 4	0.83	0.85	83.1%	23.9%	98.7%
POD 5	0.75	0.90	75.3%	18.6%	99.1%

## Discussion

AL following restorative colorectal procedures is a dreaded complication, which is often associated with significant morbidity and even mortality [11,12]. Besides early septic complications, AL could potentially prolong the length of hospital stay, increase costs, and delay planned adjuvant treatment with long-term oncological implications [13-15].

Clinical manifestations of an AL can be insidious, and sole reliance on these for timely detection may worsen an already precarious situation [13]. Therefore, early leakage detection is desirable to improve overall and oncological patient outcomes. Moreover, with the adoption of ERAS protocols in colorectal surgery, timely exclusion of postoperative complications, particularly AL, can potentially facilitate early patient discharge.

Literature suggests that the colorectal AL rate ranges between 2% and 30% [1-4], with left-sided anastomosis having a higher leak rate than right-sided anastomosis [16]. In the present study, our leak rate was 3%, consistent with previous literature [17-19]. Our relatively low leak rate could partly be explained by excluding patients undergoing emergency surgery. Messias et al. reported a leak rate of 12.2% in a cohort of patients undergoing emergency and elective colorectal resections [6]. Hoek and colleagues suggest that the low prevalence of AL might have implications for the NPV of CRP in detecting leakage after rectal cancer surgery [20].

Given the profound implications of an AL, there has been considerable research interest in improving the predictability of this unwanted phenomenon, mainly through serial measurements of various inflammatory biomarkers [1,21,22]. Although several markers have been suggested, the acute-phase reactant CRP has garnered considerable interest.

This has included recording absolute CRP levels, performing daily CRP checks postoperatively, monitoring CRP trajectory (over 50 mg/L/per day increase), and the most sensitive POD CRP cut-off value [4,8,12,20].

Our study focused on the optimal CRP cut-off value for AL exclusion. The most sensitive CRP value predictive of AL exclusion was on POD4, with an AUC of 0.90, similar to findings reported by Messias et al. (retrospective assessment of CRP levels in a cohort of 90 patients) [6].

In a subgroup meta-analysis of 10 heterogeneous studies, Rama et al. identified days three to five as the most sensitive interval during which CRP values are most predictive of AL [23], further corroborating our findings. Although Choi et al. identified POD3 CRP as being the most predictive, their findings also acknowledged that the CRP level remained high throughout days three to five in the cohort who had AL [4].

There is practical value in determining the absolute CRP cut-off value that will trigger suspicion of AL following colorectal resections. The findings of this study strongly suggest that a CRP value above 160 mg/L on POD4 is a reliable predictor of AL with sensitivity, specificity, and NPV of 85%, 83%, and 98%, respectively. Despite the low PPV value (24%) observed in this study, the practical benefit of this cut-off is highlighted by the fact that AL can be excluded with CRP values ≤160 mg/L on POD4. Therefore, such patients may be safely discharged within a clinical context if deemed appropriate.

The literature regarding the optimal CRP cut-off point for predicting AL is heterogeneous. In a meta-analysis of 11 studies, the CRP cut-off value for POD3-4 was reported to range from 94 to 190 mg/L [24]. Similarly, in a meta-analysis of 10 studies, Rama et al. estimated CRP cut-off values of 150.7 ± 30.5 and 103.5 ± 35.9 mg/L on POD3 and POD5, respectively [23].

Waterland et al. and Lagoutte et al. reported a predictive cut-off value between 123 and 125 mg/L on POD4 as a sensitive marker for AL after elective colorectal resections [25,26]. Furthermore, two studies identified the POD3 CRP value as the most predictive parameter for AL, yet the absolute values differed (163 mg/l and 140 mg/l) [27,28]. This wide variation could be attributed to the heterogeneity of the investigated cohorts, especially regarding the type of surgical resection, the timing of surgery (emergency vs. elective), the approach (open vs. laparoscopic vs. robotic), and a lack of consensus on defining an AL [6,20].

In the present study, patients with open procedures had a higher CRP value in the first three postoperative days than those undergoing laparoscopic and robotic resections. Moreover, it has been suggested that CRP levels vary between individuals based on blood loss, duration of surgery, and body mass index (BMI) [29,30]. A meta-analysis of 2,400 patients concluded that CRP levels on days three, four, and five had comparable predictive accuracy [13].

This study is not without its limitations. Firstly, our study is retrospective and open to biases, including selection bias. The study design does not allow for controls over data collection protocols, leading to potential inconsistencies in the documentation of patient demographics and operative and postoperative complications. Moreover, this study included all types of colorectal resections and anastomoses, both minimally invasive and open approaches. While these broad inclusion criteria enrich the patient population's diversity and enhance the findings' applicability across different surgical techniques, they also introduce variability that might affect postoperative CRP levels and the incidence of AL. Postoperative management, including fluids, antibiotics, and pain management, was not standardised across the study population, reflecting the diversity of practices between surgeons and healthcare units. Despite these limitations, this study's findings will contribute to our understanding of the utility of CRP in excluding AL after elective colorectal resections, aiding in clinical decision-making and patient management.

## Conclusions

In conclusion, the evidence suggests that CRP is a valuable negative predictor of AL following major colorectal surgery. This study showed an optimal cut-off value of less than 160 mg/L at POD4 as a predictor of AL exclusion. Monitoring postoperative CRP, combined with clinical assessment, can help decrease the length of hospital stay and overall cost. However, it is essential to interpret CRP levels in the context of individual patient factors and consider them alongside other clinical parameters for a comprehensive assessment.
